# Nanoelectrochemical Monitoring of pH-Regulated Reactive Oxygen and Nitrogen Species Homeostasis in Macrophages Lysosomes during Phagocytosis

**DOI:** 10.34133/research.0733

**Published:** 2025-06-05

**Authors:** Yu-Ting Qi, Rui-Xue Gao, Ying Chen, Bing-Yi Guo, Ming-Yong Wen, Christian Amatore, Wei-Hua Huang

**Affiliations:** ^1^ College of Chemistry and Molecular Sciences, Wuhan University, Wuhan, People’s Republic of China.; ^2^ Chimie Physique et Chimie du Vivant, Département de Chimie, Ecole Normale Supérieure, PSL Université, Sorbonne Université, CNRS, Paris 75005, France.; ^3^ State Key Laboratory of Physical Chemistry of Solid Surfaces, College of Chemistry and Chemical Engineering, Xiamen University, Xiamen, People’s Republic of China.; ^4^ Department of Hepatobiliary and Pancreatic Surgery, Zhongnan Hospital of Wuhan University, Wuhan, People’s Republic of China.

## Abstract

Macrophages participate in the immune system by recognizing and engulfing foreign bodies inside phagosomes, which fuse with lysosomes in their cytoplasm to form mature phagolysosomes. Lysosomes have an acidic interior and generate and release reactive oxygen and nitrogen species (ROS/RNS) to destroy the endocytosed entities. It has been previously reported that intra-lysosomal pH plays an essential role in the regulation of ROS/RNS. However, the exact regulatory mechanism remains to be elucidated. Taking advantage of the large number of active lysosomes distributed along the phagocytic lumen during frustrated phagocytosis of glass fibers by macrophages, the intensity of 4 primary ROS/RNS released fluxes (ONOO^−^, H_2_O_2_, NO, and NO_2_^−^) was monitored with platinum nanoelectrochemical sensors, thereby revealing the important role of intra-lysosomal pH on ROS/RNS fluxes after pharmacological modulations. Acidification (pH <5.0) does not alter the rate of production of ROS/RNS precursors (superoxide ions, O_2_^•−^, and parent NO) but promotes O_2_^•−^ protonation, leading to an increase of H_2_O_2_ release. In contrast, the initial production of NO, which subsequently increased the release of ONOO^−^ and NO_2_^−^, was enhanced by alkalinization (pH >6.0). The resulting increased oxidative stress was associated with massive proinflammatory cytokine release. Taken together, these results provide important information about the impact of lysosomal pH on ROS/RNS regulation.

## Introduction

As one of the specialized phagocytes, macrophages are capable of recognizing and engulfing microorganisms, pathogens, cellular debris, etc., through the expression of specific receptors on their surface and then contain the engulfed material in special vesicles called phagosomes [1–3]. These then fuse with lysosomes to form mature phagolysosomes that are equipped with an acidic lumen [1–3]. Phagolysosomes carry active pools of NADPH oxidases (NOXs) and inducible nitric oxide synthases (iNOSs) that act synergistically to produce superoxide ions (O_2_^•−^) and nitric oxide (NO), which subsequently generate 4 primary reactive oxygen species (ROS)/reactive nitrogen species (RNS) (ONOO^−^, H_2_O_2_, NO, and NO_2_^−^) that, in turn, lead to a cascade of more harmful ROS/RNS [4–6].

An increasing amount of studies have indicated that lysosomes are key players in macrophage homeostasis [7,8]. Alterations in lysosomal functions can lead to a wide range of deleterious impacts, like failure to eliminate possible toxic entities and cell waste, apoptosis, imbalance of cell signaling, and many pathological conditions [7–10]. In particular, changes in lysosomal pH have been linked to ROS/RNS production, cytokine secretion, cellular phenotype, and phagocytotic function, thereby affecting macrophage-mediated immune stress [3,8,11–13]. However, the mechanism of precise regulation of ROS/RNS generation by lysosomal pH remains unclear due to the lack of quantitative information on the exact nature and fluxes of the ROS/RNS assumed to be involved.

Nanoelectrochemical biosensors have achieved extremely sensitive and precise measurements showing elevated selectivity and outstanding spatiotemporal resolution, therefore facilitating the quantification and characterization of molecular fluxes at the level of the single cell or organelle among active cells without compromising their completeness and functions [14–18]. Under the conditions, platinum black nanoelectrodes have the unique capability of differentially and quantitatively monitoring the individual concentrations of 4 primary ROS/RNS (ONOO^−^, H_2_O_2_, NO, and NO_2_^−^) and tracking their changes over time [16,19–22]. In particular, platinum black-decorated SiC-nanowire electrochemical sensors (Pt NWSs) have proven to be extremely valuable owing to their high analytical sensitivity, their nanometric spatial resolution, and ease of construction [21,22]. They provide valuable and statistically significant dynamic information in real time.

**Fig. 1.**
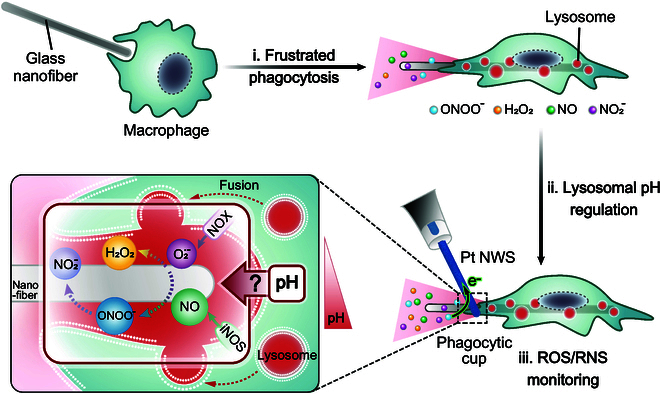
Schematic diagram describing ROS/RNS fluxes emitted at a macrophage phagocytic cup being monitored with a Pt NWS after pharmacological modulation of lysosomal pH in the frustrated phagocytosis of a glass nanofiber. Red dots shown around the phagocytotic cup represent active connected lysosomes (see Fig. 2B).

Therefore, Pt NWSs were chosen to supervise the temporal changes in the intensities of ROS/RNS fluxes secreted by single macrophages after drug-modulated lysosomal pH changes during phagocytosis of glass nanofibers (Fig. 1). Due to their high aspect ratio, glass nanofibers cannot be rapidly phagocytosed, which prevents phagocytic cups from closing and allows ROS/RNS leakage into the extracellular space [22] (step i of Fig. 1). Owing to this specific characteristic of frustrated phagocytosis and its consequences on health [22], the variations of the released fluxes of ROS/RNS were investigated via quadruple potential chronoamperometric sequences by Pt NWSs positioned at the phagocytic cups after modifying the lysosomal pH values with pharmacological agents via quadruple potential chronoamperometric sequences (step ii of Fig. 1 and Figs. S1 and S2). This allowed, for the first time, to gather quantitative data on the production of ROS/RNS by macrophages at different lysosomal pH values. Statistical analyses revealed that lysosomal acidification (pH <5.0) favored the generation of H_2_O_2_ as opposed to that of ONOO^−^ due to the increased protonation rate of O_2_^•−^. Conversely, lysosomal alkalinization (pH >6.0) was observed to stimulate iNOS expression, aggravating oxidative stress by producing substantial amounts of parent NO, hence increased RNS (ONOO^−^ and NO_2_^−^) production, and induced the release of large amounts of inflammatory factors. These quantitative data provided important insights into the role of lysosomal pH in the regulation of ROS/RNS production during macrophage immune responses.

## Results

### Importance of lysosome fusion for ROS/RNS secretion during phagocytosis

As a preliminary result, the importance of lysosome fusion with phagosomes was assessed by incubating macrophages with vacuolin-1 (Vac-1) (Figs. S1 and S3A to D). Vac-1 is a triazine-based cell-permeable compound that inhibits the integration of lysosomes into plasma membranes and compromises the function of V-ATPase (adenosine triphosphatase), preventing the secretion of lysosomal contents and resulting in an elevated lysosomal pH [23,24]. The magnitude of ROS/RNS secreted by macrophages incubated with Vac-1 was monitored with Pt NWSs (see next sections), evidencing that it was dramatically reduced to approximately one-fourth that of the control group (see below and Fig. S3E to J and Table S1). Besides, the pH in the phagocytic lumen increased relative to controls (Fig. S3D). These results proved that the fusion of lysosomes with the phagocytic lumen is a critical process during phagocytosis through the generation of ROS/RNS, which is investigated in more detail below.

### Lysosomal distribution during frustrated phagocytosis of glass fibers by macrophages

Macrophages spontaneously attempt phagocytosis of inert glass nanofibers of different lengths *L*, as shown in previous reports. Nanofibers are fully encapsulated when *L* is in the range of 10 μm or less. Conversely, for longer nanofibers, the phagocytic process cannot result in complete encapsulation of the nanofibers even after 12 to 24 h of incubation [22,25].

**Fig. 2.**
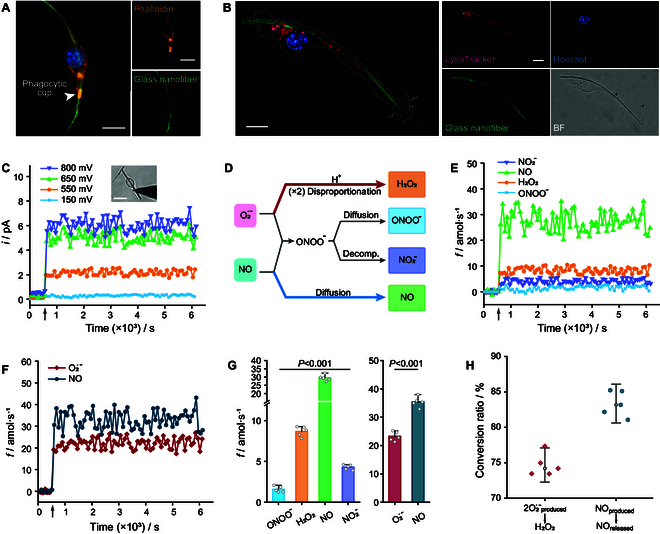
ROS/RNS leaking out the phagocytotic cups after 12 h of phagocytosis of glass nanofibers by macrophages. (A) Morphology of RAW 264.7 macrophages stained with phalloidin (orange) and Hoechst dye (blue) after 12 h of frustrated phagocytosis with glass nanofibers (green). The highlighted orange area illustrated by the white arrowhead represents the actin ring at the phagocytic cup. Scale bars, 10 μm. (B) Macrophage after 24-h phagocytosis of a glass nanofiber (green) stained with LysoTracker (red) and Hoechst dye (blue). Scale bars, 10 μm. BF, bright-field. (C) Change of the measured chronoamperometric current with time at the end of each staircase 20-s period at stepped potential values of 150 mV, 550 m, 650 m, and 800 mV versus Ag/AgCl when the Pt NWS tip was close to the phagocytic cup (inset photograph; scale bar, 20 μm) at the moment illustrated by the gray vertical arrow. (D) Illustration of the network of swift follow-up reactions involving the 2 precursors O_2_^•−^ and NO to yield the 4 primary ROS/RNS (boxes on the right). O_2_^•−^ is protonated and disproportionates into H_2_O_2_ (stoichiometry: 2O_2_^•−^ + 2H^+^ per H_2_O_2_) spontaneously (or through catalysis by superoxide dismutase within cellular bodies); O_2_^•−^ combines with NO effectively to produce ONOO^−^ (stoichiometry: 1O_2_^•−^ and 1NO per ONOO^−^) as a transient species swiftly evolving at pH <6.8 into its end-product NO_2_^−^ (whose formations also align with 1O_2_^•−^ and 1NO per NO_2_^−^). (E) Corresponding time change of the ONOO^−^ (blue), H_2_O_2_ (orange), NO (green), and NO_2_^−^ (purple) production rates at the phagocytic cup, as deduced from the currents in (C) (see Methods). (F) Time variation of the production rates of the 2 precursors O_2_^•−^ and NO at the phagocytic cup, as deduced from the data in (E), in accordance with the reaction stoichiometries shown in (D). (G) Statistical analyses (*n* = 5 macrophages) of the production rates of 4 primary ROS/RNS and their precursors O_2_^•−^ and NO at the phagocytic cup (average over 1.5 h, mean ± SEM; one-way ANOVA). (H) Conversion percentages (*n* = 5 macrophages) of the H_2_O_2_ and released NO generated from their 2 precursors O_2_^•−^ and parent NO, respectively, according to the conversion relationships of red (2O_2_^•−^_produced_ → H_2_O_2_) and blue (NO_produced_ → NO_released_) lines shown in (D).

For example, in the case of nanofibers with a mean length exceeding 70 μm, the morphology of quiescent RAW 264.7 macrophages (M0) had significant changes with apparently extended cell bodies along the fiber axes to ultimately adopt polarized shapes along the main axis of the glass nanofibers (Fig. 2A). In addition, the clearly visible actin ring of phalloidin dye appeared at the junction of the polarized macrophage with the still unengaged parts of the glass nanofibers (Fig. 2A, white arrowheads in the left photo), suggesting the formation of phagocytic cups during frustrated phagocytosis [22,25,26]. Unlike pathogens, which are easily imprisoned inside sealed phagolysosomes and broken down into small molecules, long glass nanofibers cannot be fully engulfed and are too inert to be degraded by ROS/RNS. This leads to the formation of extended unsealed phagocytic lumens from which the nanofibers protrude out of the cell. Interestingly, LysoTracker-labeled lysosomes exhibited an axial distribution along partially engulfed fibers during prolonged phagocytosis (see, e.g., Fig. 2B taken after 24 h). This phenomenon indicates that, as occurs in the process of phagolysosome formation, many lysosomes attempt to fuse with the unenclosed phagocytic lumen, thereby forming microdomains whose pH is a priori controlled by secretory lysosomes.

### Spontaneous ROS/RNS production after 12 h of frustrated phagocytosis of glass nanofibers by macrophages (controls)

To generate a statistically valid control database to compare the intensities and chemical property of ROS/RNS fluxes secreted by macrophages incubated with drugs (see below), tips of Pt NWSs were positioned at the phagocytic cup following a 12-h frustrated phagocytosis of glass nanofibers by untreated RAW 264.7 macrophages (Fig. S1). The time-dependent production rates of each primary ROS/RNS species, $f_{\text{species}}\left(t\right)$, were then monitored quantitatively using the quadruple potential chronoamperometric sequences described previously (see Methods) [19–22]. The phagocytic cup location was determined for each control experiment prior to the individual ROS/RNS monitoring. This was performed by poising the Pt NWSs at +800 mV versus Ag/AgCl to supervise all released ROS/RNS and scanning their tip along cell membranes, including the partially engulfed nanofiber, to identify the source of maximum ROS/RNS emission intensities (see Methods). Indeed, consistent with previous measurements [22], the oxidative currents monitored by Pt NWSs increased sharply when their tips reached the phagocytic cup position, evidencing the important local leakage of ROS/RNS in the process of frustrated phagocytosis (Fig. 2C).

The quadruple potential chronoamperometric sequences were then applied. According to Eqs. 1 to 4 (see Methods) and the reaction stoichiometries presented in Fig. 2D, the production rates of 4 primary ROS/RNS (Fig. 2E) and their 2 precursors O_2_^•−^ and NO (Fig. 2F) could be deduced from the currents monitored at +150, 550, 650, and 800 mV versus Ag/AgCl: $f_{{\text{ONOO}}^{-}}^{\text{Ctrl}}=1.6\pm 0.4\;\text{amol}/s$, $f_{H_{2}O_{2}}^{\text{Ctrl}}=8.7\pm 0.6\;\text{amol}/s$, $f_{\mathrm{NO}}^{\text{Ctrl}}=29.5\pm 1.9\;\text{amol}/s$, and $f_{{\mathrm{NO}}_{2}^{-}}^{\text{Ctrl}}=4.3\pm 0.3\;\text{amol}/s$, corresponding to $f_{\text{parent}\;O_{2}^{\cdot -}}^{\text{Ctrl}}=23.2\pm 1.7\;\text{amol}/s$ and $f_{\text{parent}\;\mathrm{NO}}^{\text{Ctrl}}=$ 35.4±1.8amol/s for the O_2_^•−^ and NO precursors (Fig. 2G and Tables S1 and S2). In addition to serving as controls, these data demonstrated that NO was the predominant parent ROS/RNS precursor (i.e., ca. 60% versus ca. 40% for O_2_^•−^) produced by control macrophages following a 12-h frustrated phagocytosis (Fig. S4). Significantly, during the follow-up reactions undergone by the 4 primary ROS/RNS within the phagocytic lumen (Fig. 2D), approximately 75% of the O_2_^•−^ reacted with protons to form H_2_O_2_ (note that forming 1 H_2_O_2_ consumes 2 O_2_^•−^ + 2 H^+^) (Fig. 2H and Table S2). Conversely, approximately 85% of the parent NO was released through the cup without yielding ONOO^−^ (Fig. 2H and Table S2).

### Modulation of lysosomal activity by pH during frustrated phagocytosis

**Fig. 3.**
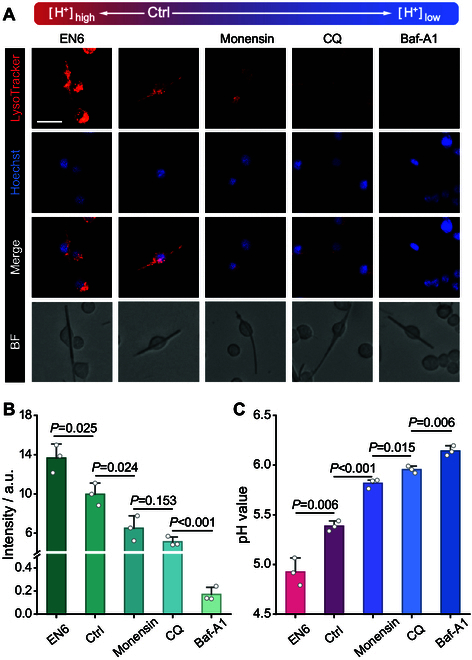
Intra-lysosomal pH values from macrophages incubated with different pharmacological agents. (A) Confocal and bright-field micrographs of RAW 264.7 macrophages stained with LysoTracker (red) and Hoechst dye (blue) following incubation with or without (Ctrl) lysosomal pH regulator (100 μM EN6, 40 μM monensin, 100 nM Baf-A1, or 20 mΜ CQ) for 2 h after 10 h of phagocytosis of glass nanofibers. Scale bar, 20 μm. (B) Statistical analysis (*n* = 90 macrophages from 3 tests) of the fluorescence intensity of LysoTracker (data are denoted as the mean ± SEM; one-way ANOVA). (C) Intra-lysosomal pH values of macrophages as figured out with the standard curve in Fig. S4 after staining with LysoSensor Yellow/Blue DND-160 (data are denoted as the mean ± SD; one-way ANOVA).

Four pharmacological agents (EN6 [27], monensin [28], chloroquine (CQ) [29], and bafilomycin A1 (Baf-A1) [30]) were employed to regulate the intra-lysosomal pH after 10 h of frustrated phagocytosis of glass nanofibers by untreated macrophages (Fig. S1), which were then incubated for 2 h with the selected drug. Importantly, bright-field microscopy revealed no remarkable changes in the phagocytic behavior of the macrophages after drug incubation (Fig. 3A). Lysotracker, an eosinophilic fluorescent probe, was used for specific fluorescent labeling of lysosomes since it exhibits an increase in fluorescence at low pH. This revealed that EN6 was effective in acidifying lysosomes in comparison to the control group, as indicated by the stronger red fluorescence (Fig. 3A and B). In contrast, monensin, CQ, and Baf-A1 induced an increasing lysosomal alkalinization in this order, as evidenced by a reduction in fluorescence intensity versus controls (Fig. 3A and B). More precise evaluations using LysoSensor confirmed that EN6 induced a decrease of intra-lysosomal pH from 5.39 to 4.92, whereas monensin, CQ, and Baf-A1 increased it to 5.82, 5.96, and 6.14, respectively (Fig. 3C). Besides, a short drug incubation period of 2 h does not significantly alter the characteristics of lysosomal distribution along the nanofibers (Fig. 3A). The above results demonstrated that incubation with this panel of drugs was effective in modulating the intra-lysosomal pH in both directions versus controls.

### Effect of lysosomal acidification on ROS/RNS production by macrophages

**Fig. 4.**
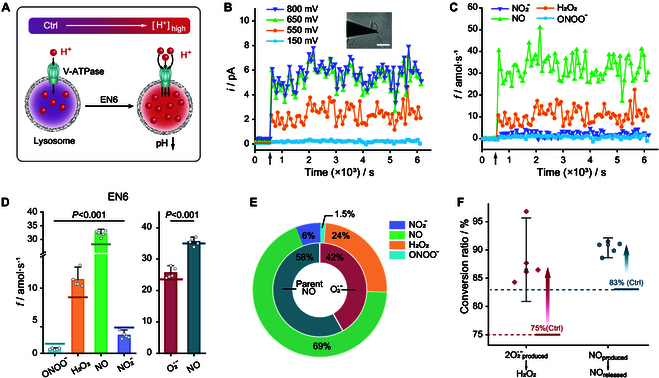
ROS/RNS production of macrophage after lysosomal acidification. (A) Schematic diagram of EN6 acidification of the lysosomal lumen due to the accelerated proton pumping by the vesicular H^+^-ATPase (V-ATPase). (B) Time change of the chronoamperometric current at stepped potential values indicated after incubation of macrophages with 100 μM EN6 for 2 h, after 10 h of frustrated phagocytosis, when the Pt NWS tip was near the unsealed phagocytic cup (inset photograph; scale bar, 20 μm) at the moment illustrated by the gray vertical arrow. (C) Corresponding time variation of the ONOO, H_2_O_2_, NO, and NO_2_^−^ production rates at the phagocytic cup, as deduced from the currents in (B). (D) Statistical analyses (*n* = 5 macrophages) of the production rates of the 4 primary ROS/RNS and their precursors O_2_^•−^ and NO at the phagocytic cup (data are denoted as the mean ± SEM; one-way ANOVA). The solid lines in each ROS/RNS corresponding to the bar colors represent the average production rates of the 4 primary ROS/RNS and their precursors of untreated group (Ctrl) as illustrated in Fig. 2G. (E) Relative proportions of the 4 primary ROS/RNS and their precursors O_2_^•−^ and NO, as deduced from the statistical data in (D). (F) Percentages of initially produced O_2_^•−^ converted into H_2_O_2_ (2O_2_^•−^_produced_ → H_2_O_2_) and the percentage of released NO (NO_produced_ → NO_released_) after macrophage incubation with EN6. The dashed lines represent the average conversion rates of control group (Ctrl) in Fig. 2H (data are denoted as the mean ± SEM; one-way ANOVA).

Vesicular H^+^-ATPase (V-ATPase) is an evolutionarily conserved adenosine triphosphate (ATP)-driven rotary proton pump facilitating the pumping of protons into the lysosomal interior, thereby creating an acidic environment therein [31]. EN6 covalently binds to the ATPV1A subunit of V-ATPase, which triggers to inhibit mTORC1 signaling and raises the catalytic activity of V-ATPase, thereby exacerbating lysosomal acidification [27] (Fig. 4A).

As the nanosensor tip reached the phagocytic cup of the EN6-treated macrophages, the current immediately increased as in the control group (Fig. 4B). However, the production rates of the 4 primary ROS/RNS were affected to discrepant extents compared to the controls. A significant increase in the rate of H_2_O_2_ production (+30%), a moderate one for released NO (+8.5%), and important decreases in ONOO^−^ (−50%) and NO_2_^−^ (−35%) productions were observed (Fig. 4C and D): $f_{{\text{ONOO}}^{-}}^{\mathrm{EN}6}=0.7\pm 0.1\;\text{amol}/s$, $f_{H_{2}O_{2}}^{\mathrm{EN}6}=11.3\pm 1.3\;\text{amol}/s$, $f_{\mathrm{NO}}^{\mathrm{EN}6}=32.1\pm 1.0\;\text{amol}/s$, and $f_{{\mathrm{NO}}_{2}^{-}}^{\mathrm{EN}6}=2.8\pm 0.5\;\text{amol}/s$. In fact, the amount of H_2_O_2_ released versus that of all ROS/RNS increased from 20% to 24%, while those of ONOO^−^ and NO_2_^−^ decreased from 4% and 10% to 1.5% and 6%, respectively (Fig. 4E). It is important to note that these substantial changes did not result from significant differences in the mean production rates of the parents O_2_^•−^ and NO precursors (Fig. 4D and Fig. S6): $f_{\text{parent}\;O_{2}^{\cdot -}}^{\mathrm{EN}6}=25.6\pm 1.7\;\text{amol}/s$ (+10%) and $f_{\text{parent}\;\mathrm{NO}}^{\mathrm{EN}6}=35.6\pm 1.2\;\text{amol}/s$ (<1%). These statistically significant results demonstrated that lysosomal acidification increased the protonation rate of the parent O_2_^•−^, favoring its disproportionation into H_2_O_2_ over its reaction with NO to form ONOO^−^ (Fig. 4F).

### Effect of lysosomal alkalinization on ROS/RNS production by macrophages

**Fig. 5.**
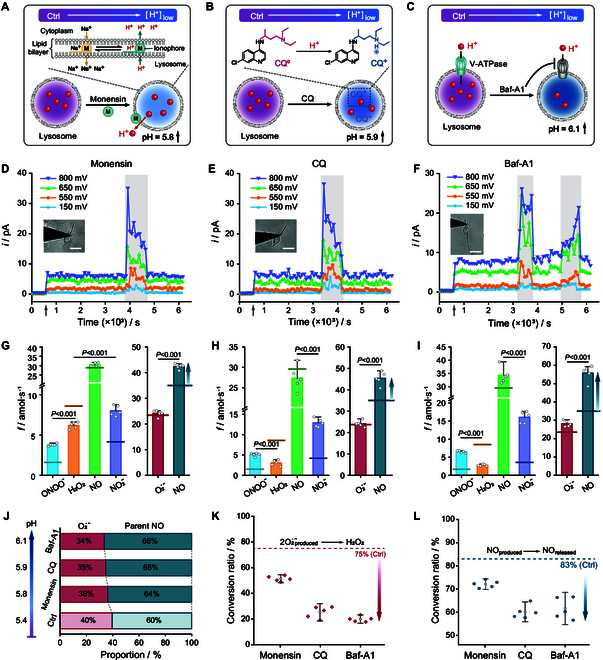
ROS/RNS production of macrophage after lysosomal alkalization. (A to C) Schematic diagrams of the mechanism of lysosomal alkalinization by monensin (A), CQ (B), and Baf-A1 (C). (D to F) Time changes of the chronoamperometric currents at stepped potential values after macrophage incubation for 2 h, after 10 h of frustrated phagocytosis, with 40 μM monensin (D), 20 mΜ CQ (E), and 100 nM Baf-A1 (F) when the Pt NWS tip was near the phagocytic cup (inset photographs; scale bar, 20 μm) at the moment illustrated by the gray vertical arrow. (G to I) Statistical analyses (*n* = 5 macrophages) of the production rates of the 4 primary ROS/RNS and their precursors O_2_^•−^ and NO at the phagocytic cups with incubation of 40 μM monensin (G), 20 mΜ CQ (H), and 100 nM Baf-A1 (I) (data are denoted as the mean ± SEM; one-way ANOVA). The horizontal solid lines indicated in (G) to (I) for each ROS/RNS bar colors represent the average production rates of the 4 primary ROS/RNS and their precursors of untreated group (Ctrl) as illustrated in Fig. 2G. (J) Relative proportions of the initially produced precursors O_2_^•−^ and NO and pH value after incubation with monensin, CQ, and Baf-A1. (K and L) Percentages of initially produced O_2_^•−^ converted into H_2_O_2_ (2O_2_^•−^_produced_ → H_2_O_2_) (K) and the percentage of released NO (NO_produced_ → NO_released_) (L) after macrophage incubation of monensin, CQ, and Baf-A1. The dashed lines represent the average conversion rates measured for the control group (Ctrl) in Fig. 2H (data are denoted as the mean ± SEM; one-way ANOVA).

To investigate the changes in ROS/RNS production upon increasing lysosomal alkalinization, cells were incubated with different pharmacological agents (monensin, CQ, and Baf-A1) in 3 separate series of experiments. Monensin is a monovalent ion-selective ionophore that is soluble in the lipid component of the membrane and freely shuttles across the aqueous membrane interface, causing decrease in intra-lysosomal acidity by promoting trans-membrane exchange of sodium ions and protons [28] (Fig. 5A). CQ is one of the most commonly prescribed medications to treat chronic and severe inflammatory diseases [29]. At neutral pH, CQ is uncharged and is thus prone to diffuse freely across the lysosome membrane to be protonated therein, thereby raising the intra-lysosomal pH [29] (Fig. 5B). Baf-A1 is a macrolide antibiotic that has been investigated for its potential as anticancer agent based on its capacity to inhibit cell proliferation and tumor growth by acting as selective and efficient V-ATPase inhibitor [30] (Fig. 5C). Although the 3 agents act by different mechanisms, they all cause the lysosomal pH to rise, reaching levels exceeding 5.5. They have therefore been selected to avoid false positives.

The protocols used in each case were identical to that used for EN6, i.e., 2-h incubation with the drug after 10 h of frustrated phagocytosis of glass nanofiber. Compared to controls, macrophages stimulated with all each lysosomal alkalinizing drug exhibited significantly decreased rates of H_2_O_2_ production and markedly increased rates of ONOO^−^ and NO_2_^−^ production (Fig. S7A, C, and E). In addition, the rate of the parent NO production increased markedly following incubation of the cells with each alkalinizing drug, whereas the production rate of O_2_^•−^ remained almost unchanged (Fig. S7B, D, and F). Notably, as shown in Fig. 5D to F, 1 or 2 sudden current bursts of ca. 10- to 15-min duration were randomly observed over periods of ca. 2 h of monitoring (see the gray intervals in Fig. 5D to F and the discussion below). In the absence of any indication as to their cause, these sudden bursts of current were excluded from the following statistical analyses. The latter showed that after lysosomal alkalinization by each of the 3 drugs, the production rates of H_2_O_2_ decreased to approximately 70% (monensin), 40% (CQ), or 30% (Baf-A1) of those in the control group; conversely, the production rates of ONOO^−^ and NO_2_^−^ were approximately 2, 3, and 4 times higher, respectively, than in the control group: $f_{{\text{ONOO}}^{-}}^{\text{monensin}}=3.8\pm 0.2\;\text{amol}/s$, $f_{H_{2}O_{2}}^{\text{monensin}}\;=\;6.2\pm 0.4\;\text{amol}/s$, $f_{\mathrm{NO}}^{\mathrm{EN}6}\;=\;30.6\;\pm \;1.1\;\text{amol}/s$, and $f_{{\mathrm{NO}}_{2}^{-}}^{\mathrm{EN}6}\;=\;8.0\;\pm \;0.7\;\text{amol}/s$; $f_{{\text{ONOO}}^{-}}^{\mathrm{CQ}}=5.1\pm 0.4\;\text{amol}/s$, $f_{H_{2}O_{2}}^{\mathrm{CQ}}=3.1\pm 0.7\;\text{amol}/s$, $f_{\mathrm{NO}}^{\mathrm{CQ}}=$ 27.3±3.0amol/s, and $f_{{\mathrm{NO}}_{2}^{-}}^{\mathrm{CQ}}=12.9\pm 1.0\;\text{amol}/s$; $f_{{\text{ONOO}}^{-}}^{\mathrm{Baf}-A1}=$ 6.5±0.3amol/s, $f_{H_{2}O_{2}}^{\mathrm{Baf}-A1}\;=\;2.8\;\pm \;0.3\;\text{amol}/s$, $f_{\mathrm{NO}}^{\mathrm{Baf}-A1}=$ 34.2±3.1amol/s, and $f_{{\mathrm{NO}}_{2}^{-}}^{\mathrm{Baf}-A1}=16.1\pm 1.6\;\text{amol}/s$ (Fig. 5G to I and Table S1). Interestingly, the production rate of parent O_2_^•−^ remained almost unchanged compared to the control group, while that of NO production showed a significant increase, reaching approximately 1.2 (monensin), 1.3 (CQ), and 1.5 (Baf-A1) times the level observed in the control group: $f_{\text{parent}\;O_{2}^{\cdot -}}^{\text{monensin}}=24.1\pm 1.2\;\text{amol}/s$ and $f_{\text{parent}\;\mathrm{NO}}^{\text{monensin}}\;=\;42.4\pm 1.0\;\text{amol}/s$; $f_{\text{parent}\;O_{2}^{\cdot -}}^{\mathrm{CQ}}\;=\;24.5\pm 1.5\;\text{amol}/s$ and $f_{\text{parent}\;\mathrm{NO}}^{\mathrm{CQ}}\;=\;45.3\;\pm \;3.0\;\text{amol}\;/\;s$; $f_{\text{parent}\;O_{2}^{\cdot -}}^{\mathrm{Baf}-A1}\;=\;28.2\;\pm \;1.6\;\text{amol}\;/\;s$ and $f_{\text{parent}\;\mathrm{NO}}^{\mathrm{Baf}-A1}=55.8\pm 4.3\;\text{amol}/s$ (Fig. 5G to I and Table S1). Given that the productions of NO and O_2_^•−^ are known to be primarily regulated by iNOS and NOX [32,33], these changes were tentatively attributed to variations in the expression of both enzymes under the influence of the 3 pharmacological agents. The validity of this hypothesis was tested using fluorescence staining (Figs. S8A and S9A). The corresponding statistical results confirmed this hypothesis by showing that iNOS expression increased after incubation of cells with each lysosomal alkalinizing drug, whereas NOX expression did not show significant changes (Figs. S8B and S9B).

These results indicated that lysosomal alkalinization is associated with increased iNOX expression, hence to a larger production of parent NO than in controls (Fig. 5J). NOX expression was not visibly affected, but due to the lack of protons, the generated parent O_2_^•−^ amounts reacted more rapidly with NO to yield ONOO^−^, which partially decomposed into NO_2_^−^. This results in an approximately 50% and 20% decrease in the conversion of initially generated O_2_^•−^ into H_2_O_2_ and that of released NO, respectively (Fig. 5K and L and Table S2). Consequently, after incubation of the cells with each of the 3 alkalinizing drugs, the percentage of H_2_O_2_ gradually decreased with increasing pH, while those of ONOO^−^ and NO_2_^−^ increased (Fig. S10).

At this time, a definitive explanation for the bursts observed in Fig. 5D to F cannot be provided due to their brief and random nature, although it is apparent that they correspond to highest concentrations of the same ROS/RNS, albeit in different proportions than before or after their occurrence. However, given that LysoTracker staining indicated that acidic organelles accumulated at the boundary of the phagocytic lumen along the nanofibers axes (Fig. 2B), and that previous reports indicated that lysosomal alkalinization is a contributing factor in macrophage–lysosomal fusion events [2,3,34,35], it is reasonable to speculate that these bursts could represent the rapid accidental ejection of a vesicle, such as a lysosome or a late endosome, in the extracellular space at the exit of the phagocytotic cup. This would then cause a sudden dramatic increase in ROS/RNS levels as the vesicle contents are rapidly released upon impact with the nanoelectrochemical sensor tip [36–38].

## Discussion

Phagocytes are essential immune defense cells. They function by eliminating extracellular viruses, bacteria, and cellular debris through phagocytosis. This process occurs by encapsulating them into phagosomes, which subsequently fuse with lysosomes in the macrophage cytoplasm to form phagolysosomes where the trapped entities are degraded via the generation of ROS/RNS [2,3]. Lysosomes serve as metabolic sensors and signaling platforms, and their pH plays a central role in regulating immunometabolic reprogramming processes in immune cells [8,11,13]. Commonly, the pH of lysosomes varies from 4.5 to 5.0 and this is maintained mainly by V-ATPases located on the lysosomal membrane, which pump protons from the cytoplasm into the lysosomal lumen [1–3,31].

**Fig. 6.**
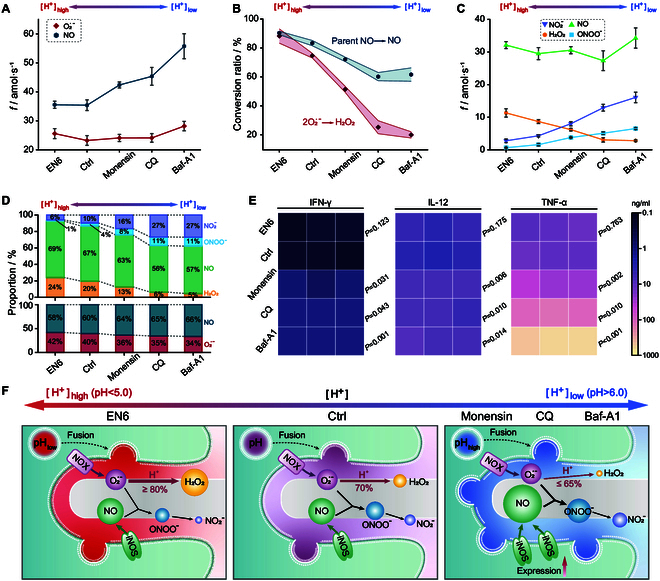
Regulation of ROS/RNS production by different pH of lysosome. (A) Production rates of the ROS/RNS precursors O_2_^•−^ and NO at different intra-lysosomal pH (data are shown as the mean ± SEM; one-way ANOVA). (B) Percentages of initially produced O_2_^•−^ converted into H_2_O_2_ (2O_2_^•−^_produced_ → H_2_O_2_) as a function of the drug alerting on the pH; for NO, the percentage of released NO is given relatively to its generated quantities (NO_produced_ → NO_released_) (data are shown as the mean ± SEM; one-way ANOVA). (C) Production rates of the 4 primary ROS/RNS at different intra-lysosomal pH (data are denoted as the mean ± SEM; one-way ANOVA). (D) Relative proportions of the 4 primary ROS/RNS and their precursors O_2_^•−^ and NO at different intra-lysosomal pH. (E) Heatmap of the cytokine array of macrophages after, or without, incubation of different pharmacological agents after 24 h (*n* = 3 biologically independent samples; comparison of remarkable disparities between groups of control and pharmacological agents; mean ± SD; one-way ANOVA). The 3 columns for each category (namely, IFN-γ, IL-12, and TNF-α) exhibit the color results of 3 discrepant parallel tests. (F) Graphical summarization of potential mechanisms consistent with the regulation of ROS/RNS production induced at different intra-lysosomal pH. Relative diameters of color patches regarding the 4 ROS/RNS match their relative detected quantities, while the thicknesses of reaction arrows stand for the relative kinetic magnitudes of the reaction rates emerging in phagolysosome lumen.

In this study, nanoelectrochemical sensors were used to delve into the function of lysosomal pH in regulating ROS/RNS generation after lysosomal–phagosomal fusion during frustrated phagocytosis of long (ca. 70 μm) glass nanofibers [22]. Conversely, a significant reduction in ROS/RNS production following the inhibition of lysosomal fusion with phagocytic lumens was observed after incubating RAW 264.7 immune macrophages with Vac-1 (Fig. S3). During normal fusion, lysosomal pH was involved in regulating the yield of ROS/RNS precursors (O_2_^•−^ and NO). The NO production was found to be drastically dependent on the expression and activation of iNOS [9]. Fluorescence staining experiments showed that iNOS expression increased with lysosomal alkalinization (Fig. S8), being consistent with literature reports [39,40]. This was in full agreement with the increase in NO production compared to controls as monitored by Pt NWSs when intra-lysosomal pH was raised above 5.5 after incubation of RAW 264.7 cells with specific drugs (Fig. 6A). The significant rise in NO production observed following incubation with CQ and Baf-A1 may be attributed to the enhancement of iNOS activation. CQ is associated with the stimulation of protein kinase C phosphorylation [41], while Baf-A1 is linked to an increase in the production of inflammatory cytokines [42] (more detail below), so both mechanisms may ultimately contribute to the activation of iNOS. Conversely, O_2_^•−^ production remained stable with pH compared to untreated cells, being consistent with the unchanged NOX expression observed by fluorescence measurements (Fig. S10).

The conversion of initially produced O_2_^•−^ and NO into other types of ROS/RNS at various lysosomal pH was quantitatively monitored by Pt NWSs. Under conditions of lysosomal acidification (pH 5.0, after 2 h of EN6 incubation), the disproportionation of O_2_^•−^ into H_2_O_2_ was favored over its coupling with NO to generate ONOO^−^ (Fig. 6B), although O_2_^•−^ production was only slightly decreased. Notably, the disproportionation of O_2_^•−^ into H_2_O_2_ tends to stabilize near pH 6.0 [43], contributing to a slowdown in the changes in conversion rates between the CQ and Baf-A1 treatments. Conversely, in line with a highest NO production and its increased stability with increasing pH (pH >5.5 after incubation with monensin, CQ, or Baf-A1), the ONOO^−^ yield was increased (Fig. 6C) [44,45]. Interestingly, the relative yield of NO_2_^−^ versus ONOO^−^ production decreased from about 4 in acidic pH (EN6) to 2.5 (Baf-A1) (see Fig. 6C and Table S1), consistent with the increased stability of ONOO^−^ as pH is increased [46,47]. The percentages of the 4 ROS/RNS and their precursors, O_2_^•−^ and NO, followed similar trends when the intra-lysosomal pH was varied (Fig. 6D).

The present series of results confirmed that the intra-organelle pH is a key factor in controlling inflammatory responses while regulating homeostatic and inflammatory programs [11,12]. To reinforce the significance of the above results, the drug-mediated release of pro-inflammatory cytokines [interferon-γ (IFN-γ), interleukin-12 (IL-12), and tumor necrosis factor-α (TNF-α)] was investigated at different lysosomal pH values. Statistical data obtained with the nanoelectrochemical sensors indicated that acidification had no significant effect on the yield of pro-inflammatory factors relative to controls, whereas the alkalization significantly increased the cytokines levels (Fig. 6E), in agreement with previous reports [39,40]. In particular, compared to controls, the strong alkalization of the intra-lysosomal domain achieved after 2-h Baf-A1 incubation increased the yield of IFN-γ, IL-12, and TNF-α through approximately 6-, 4-, and 40-fold, respectively (Fig. 6E). This confirmed that lysosomal alkalinization also stimulated the secretion of pro-inflammatory mediators, thereby enhancing their protective role against viruses, bacteria, and malignant cells [39,40,48,49].

In summary, an electrochemical nanosensor was used to provide critical quantitative perceptions of the function of lysosomal pH in modulating macrophage-based immune reactions by fine-tuning ROS/RNS composition and production (Fig. 6F), as well as pro-inflammatory cytokine production (Fig. 6E). On the one hand, lysosomal acidification (pH <5.0) was shown to accelerate the overall disproportionation of NOX-generated O_2_^•−^, resulting in an increase in H_2_O_2_ release. On the other hand, its alkalinization (pH >6.0) has been shown to enhance iNOX expression, thereby increasing the production of NO, and thus of the highly reactive ONOO^−^, by favoring NO and O_2_^•−^ coupling. Notably, this was shown to be accompanied by an exacerbation of the release of pro-inflammatory factors, thereby enhancing the effect of oxidative stress.

By offering new important perceptions of the spectacular function of lysosomal pH in modulating immune reactions, this series of quantitative results might promote the progress of subsequent immunotherapeutic strategies for treating illnesses, like autoimmune disorders, inflammatory conditions, and cancer, whenever they involve disruption of lysosomal pH homeostasis.

## Methods

### Preparation of Pt NWSs

Mechanical procedures of Pt NWS preparation were the same as those depicted in [21,22]. Briefly, SiC nanowires (ca. 200 nm diameter) self-coated with high-density Pt nanoparticles (SiC@Pt NWs) were added dropwise to the center of a glass sheet and mildly heated to evaporate water. The glass sheet was separated into 2 sections to accept a partial protrusion of NWs over the glass slide edges. Subsequently, each Pt NWs was inserted cautiously into a fabricated glass micropipette holder full of liquid metal and wax to prepare one Pt NWS sensor showing the protruding length of 5 μm.

### Cell experiments

Prior to any assay, the RAW 264.7 cells (Pricella Biotechnology Co. Ltd.) were incubated on small culture dishes (35 mm in diameter; Jet Biofil). The cellular experimental process is depicted in Fig. S1. After cell adhesion, the RAW 264.7 cells were cultured with the addition of 50 μg/ml glass nanofibers for 10 h to activate macrophages from M0 type to polarized type. Subsequently, the polarized macrophages were incubated with different pharmacological pH regulators (100 μM EN6, 40 μM monensin, 100 nM Baf-A1, 20 mΜ CQ, and 10 μM Vac-1) for 2 h to modulate the change in lysosomal pH.

### Imaging

RAW 264.7 cells after 12 h of frustrated phagocytosis of fluorescein isothiocyanate (FITC)-labeled glass nanofibers were immobilized with 4% paraformaldehyde for 10 min and permeabilized with 0.1% Triton X-100 for 15 min. In addition to fixed and permeated M0, polarized RAW 264.7 cells were cultivated with 555-phalloidin (dilution 1:40; UElandy, Suzhou China) and Hoechst 33342 (dilution 1:1000; Sigma-Aldrich) for 30 min. Confocal microphotographs were captured promptly using a Zeiss confocal microscope (LSM900).

RAW 264.7 cells following a 12-h frustrated phagocytosis of FITC-labeled glass nanofibers were cultured with 75 nM LysoTracker (Thermo Fisher) solution and 5 μg/ml Hoechst for 25 min. Subsequently, bright-field and confocal microphotographs were then recorded with a Zeiss confocal microscope (LSM900) as soon as possible.

Fixed and permeabilized RAW 264.7 cells were labeled with iNOS monoclonal fluorescent antibody (Alexa Fluor 488) (dilution 1:25; Thermo Fisher) at 4 °C overnight. NOX2 polyclonal antibody (dilution 1:100; Thermo Fisher) was incubated with cells at 4 °C overnight. FITC-conjugated goat anti-rabbit immunoglobulin G (IgG) (dilution 1:100; BOSTER), 555-phalloidin, and Hoechst 33342 were incubated with cells for 60 min. Confocal microphotographs were captured promptly with a Zeiss confocal microscope (LSM900).

### Measurement of lysosomal pH

LysoSensor Yellow/Blue is a ratiometric probe whereby the proportion of light excited at 340 to 380 nm is in proportion to lysosomal pH, which is determined through a calibration curve. To formulate a standard curve (see Fig. S5), the M0 macrophages with LysoSensor were equalized by cultivating them in a set of MES calibration buffers at pH values from 3.5 to 8.0 for 10 min at 37 °C. After that, the M0 macrophages were analyzed through a microplate reader. Fluorescence emissions were gathered at 440 and 540 nm for excitation at 340 and 380 nm, separately. RAW 264.7 cells with or without drug incubation were incubated with 5 μM LysoSensor Yellow/Blue DND-160 for 10 min at 37 °C and then rinsed thrice with phosphate-buffered saline (PBS). Next, pH values of different groups were analyzed through a microplate reader with the fluorescence emissions gathered at 440 and 540 nm, respectively.

### Cytokine and cytotoxicity detection

RAW 264.7 macrophages were cultured in 24-well plates at a density of 2.5 × 10^4^ cells/cm^2^ for the detection of cytokines and cytotoxicity. After incubating for 24 h with or without lysosomal pH regulators (100 μM EN6, 40 μM monensin, 100 nM Baf-A1, 20 mM CQ, and 10 μM Vac-1), the supernatants were collected to measure TNF-α (Neobioscience Technology Co. Ltd.), IL-12 ((Neobioscience), and IFN-γ (Neobioscience) levels using enzyme-linked immunosorbent assay (ELISA) kits. The assays were conducted with a multimode dispenser (Epoch, BioTek) following the manufacturer’s protocol.

### Quantitative characterization of individual ROS/RNS fluxes

Methods of quantitative monitoring of ROS/RNS have been reported in our previous work. Each primary ROS/RNS (ONOO^−^, H_2_O_2_, NO, and NO_2_^−^) secreted during phagocytosis is electrochemically oxidizable upon application of a specific potential (+150, 550, 650, and 800 mV versus Ag/AgCl) to the SiC@Pt NWS, in accordance with the voltammetric oxidation order of ROS/RNS [21,22]. Given that electrochemical currents are additive, the amperometric currents monitored at each of the 4 aforementioned potentials, detected in sequence via the periodic potential-step progression, are given at any given time *t* by the following equation [21,22]:

$$i_{150\;\text{mV}}\left(t\right)=i_{{\text{ONOO}}^{-}}\left(t\right)+0.1i_{H_{2}O_{2}}\left(t\right)$$ (1)

$$i_{550\;\text{mV}}\left(t\right)=i_{{\text{ONOO}}^{-}}\left(t\right)+i_{H_{2}O_{2}}\left(t\right)+0.1i_{\mathrm{NO}}\left(t\right)$$ (2)

$$i_{650\;\text{mV}}\left(t\right)=i_{{\text{ONOO}}^{-}}\left(t\right)+i_{H_{2}O_{2}}\left(t\right)+i_{\mathrm{NO}}\left(t\right)$$ (3)

$$i_{800\;\text{mV}}\left(t\right)=i_{{\text{ONOO}}^{-}}\left(t\right)+i_{H_{2}O_{2}}\left(t\right)+i_{\mathrm{NO}}\left(t\right)+i_{{\mathrm{NO}}_{2}^{-}}\left(t\right)$$ (4)

where $i_{\text{potential}}\left(t\right)$ is the total current determined at each chosen potential at time t, while $i_{\text{species}}\left(t\right)$ is the individual limiting plateau current of the named species that would be viewed at the identical time t if this species only existed at the identical concentration under the identical conditions. The independently recorded voltammetric oxidation waves for the 4 primary ROS/RNS indicated that those of ONOO^−^ and H_2_O_2_ were not fully distinct and gave rise to a partial overlap between the plateau of the ONOO^−^ wave and the foot of the H_2_O_2_ one (Fig. S2). The same situation occurred for the plateau of H_2_O_2_ and foot of NO waves. This led to added contributions due to the foot currents of H_2_O_2_ (Eq. 1) and NO (Eq. 2) into the total detected currents at +150 mV and +500 mV, respectively, which are accounted by the coefficients in Eqs. 1 and 2.

All $i_{\text{potential}}$ values were documented at the end of 20-s potential steps, and each individual plateau current value for each species could be obtained by solving the linear Eqs. 1 to 4 [22]. Subsequently, the time variations of each individual current, $i_{\text{species}}\left(t\right)$, could be determined by solving the system of Eqs. 1 to 4. Therefore, the time-dependent production rates of each ROS/RNS species, $f_{\text{species}}(t)$, could be calculated based on these currents, $f_{\text{species}}(t)$, in accordance with Faraday’s law: $f_{\text{species}}\left(t\right)=\frac{i_{\text{species}}\left(t\right)}{z_{\text{species}}F}$. Here, the Faraday constant is represented by F, and $z_{\text{species}}$ denotes the electron stoichiometry involved in the electrochemical oxidation of the species at Pt-black electrodes $\left(z_{H_{2}O_{2}}=z_{{\mathrm{NO}}_{2}^{-}}=2;z_{{\text{ONOO}}^{-}}=z_{\mathrm{NO}}=1\right)$ [22].

### Amperometric data acquisition and analysis

All amperometric measurements and data acquisition and analysis have been reported in our previous work [21,22]. The amperometric traces were recorded using a 2-electrode electrochemical system paired with a patch-clamp amplifier (EPC-10 HEKA Electronics, Germany) across a range of selected potentials (150, 550, 650, and 800 mV versus Ag/AgCl), and amperometric traces were sampled at a frequency of 1 Hz [21,22].

The method for localization of the “phagocytic cups” is similar to that reported in the [22]. Pt NWSs were scanned near the macrophages’ membrane surface following the partially captured glass fiber axis with 2-μm steps. At each site, currents were documented during 20 s at +800 mV versus Ag/AgCl and averaged to determine the average quantity of total ROS/RNS secreted at this point. The location exhibiting the maximum current of ROS/RNS release is the position of phagocytic cup.

The protocol of quadruple potential chronoamperometric sequences was executed as reported in [21,22]. The currents were sampled at the end of each 20-s-long potential step to minimize the charging current contributions. A pre/post-calibration was performed by locating the electrode far from the cell and cycling its potential for 30 min before and after each test. Then, any shift in the baseline was subtracted from the recorded reaction to generate the final chronoamperograms.

The ROS/RNS release fluxes were documented over 1.5 h with the nanoelectrode placed on the site of the phagocytic cup by an individual macrophage. Following subtraction of the baseline (current intensity values for M0 cells, undisplayed data), 70 data points were documented for each primary ROS/RNS and averaged into a value of ROS/RNS leakage from an individual cell.

Original amperometric data were collected with “PatchMaster” (v2x90.3 version) and analyzed by “Origin 2021 Graphing & Analysis”.

## Data Availability

The data are available from the corresponding author on reasonable demand.
