# Supplementary material for: Nanoelectrochemical Monitoring of pH-Regulated Reactive Oxygen and Nitrogen Species Homeostasis in Macrophages Lysosomes during Phagocytosis
Source: Research (Wash D C). 2025 Jun 5;8:0733. doi: 10.34133/research.0733 (PMC12139194; doi:10.34133/research.0733)
Supplement: Supplementary 1 — Figs. S1 to S10 Tables S1 and S2 [file research.0733.f1.docx]

***Supplementary Materials for***

**Nanoelectrochemical Monitoring of pH-Regulated ROS/RNS Homeostasis in Macrophages Lysosomes During Phagocytosis**

Yu-Ting Qi^1^, Rui-Xue Gao^1^, Ying Chen^1^, Bing-Yi Guo^1^, Ming-Yong Wen^1^, Christian Amatore^3,4,*^, Wei-Hua Huang^1,2,*^

^1^College of Chemistry and Molecular Sciences, Wuhan University, Wuhan, People’s Republic of China

^2^Department of Hepatobiliary and Pancreatic Surgery, Zhongnan Hospital of Wuhan University, Wuhan, People’s Republic of China

^3^Chimie Physique et Chimie du Vivant, Département de Chimie, Ecole normale supérieure, PSL Université, Sorbonne Université, CNRS, 24 rue Lhomond, Paris 75005, France

^4^State Key Laboratory of Physical Chemistry of Solid Surfaces, College of Chemistry and Chemical Engineering, Xiamen University, Xiamen, People’s Republic of China

*Address correspondence to: christian.amatore@ens.psl.eu and whhuang@whu.edu.cn

**SUPPLEMENTARY FIGURES**

**
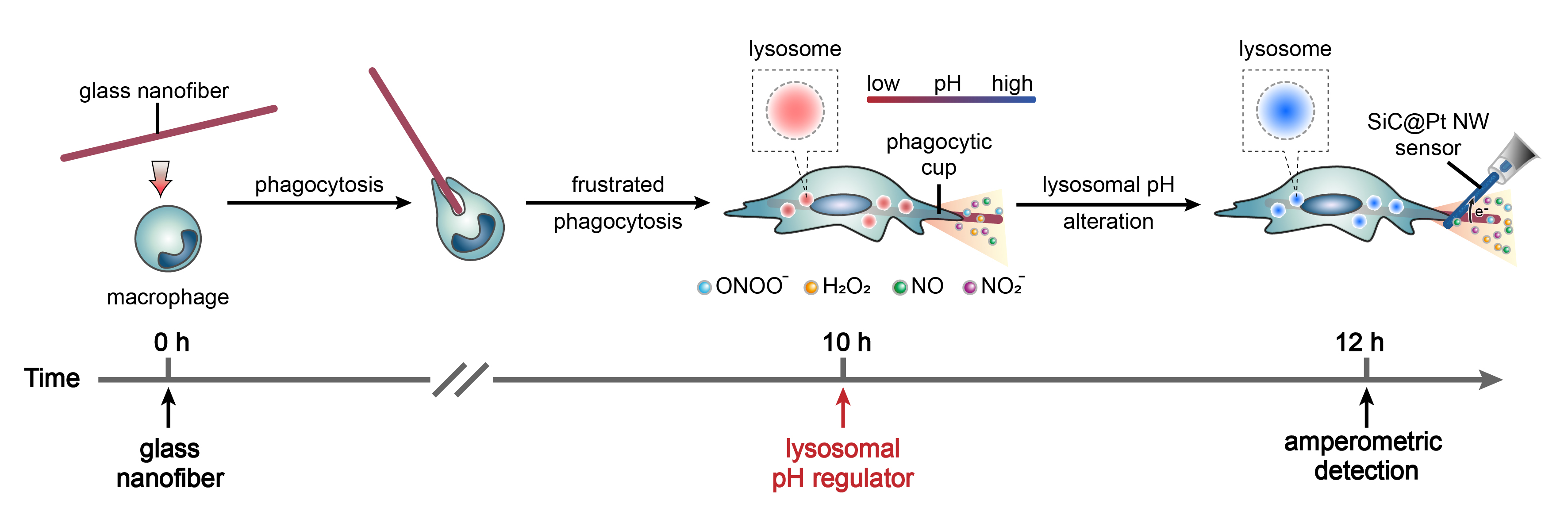
**

**Fig. S1. Frame time** **sequence of cell experiments and amperometric detection.** See section 1.4 for experimental details.


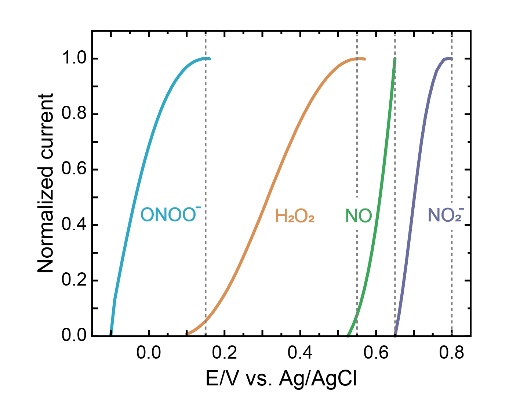


**Fig. S2. Voltammetric oxidation of ONOO^-^ (1 mM, pH=10.0), H_2_O_2_ (1 mM, pH=7.4), NO (1 mM, pH=7.4) and NO_2_^-^ (1 mM, pH=7.4) in PBS solution as recorded with the SiC@Pt NWS**. Currents were normalized to their maximum plateau value to help comparison, and vertical dashed lines indicate the optimal detection potentials for quantifying each ROS/RNS species (see Methods section).

**
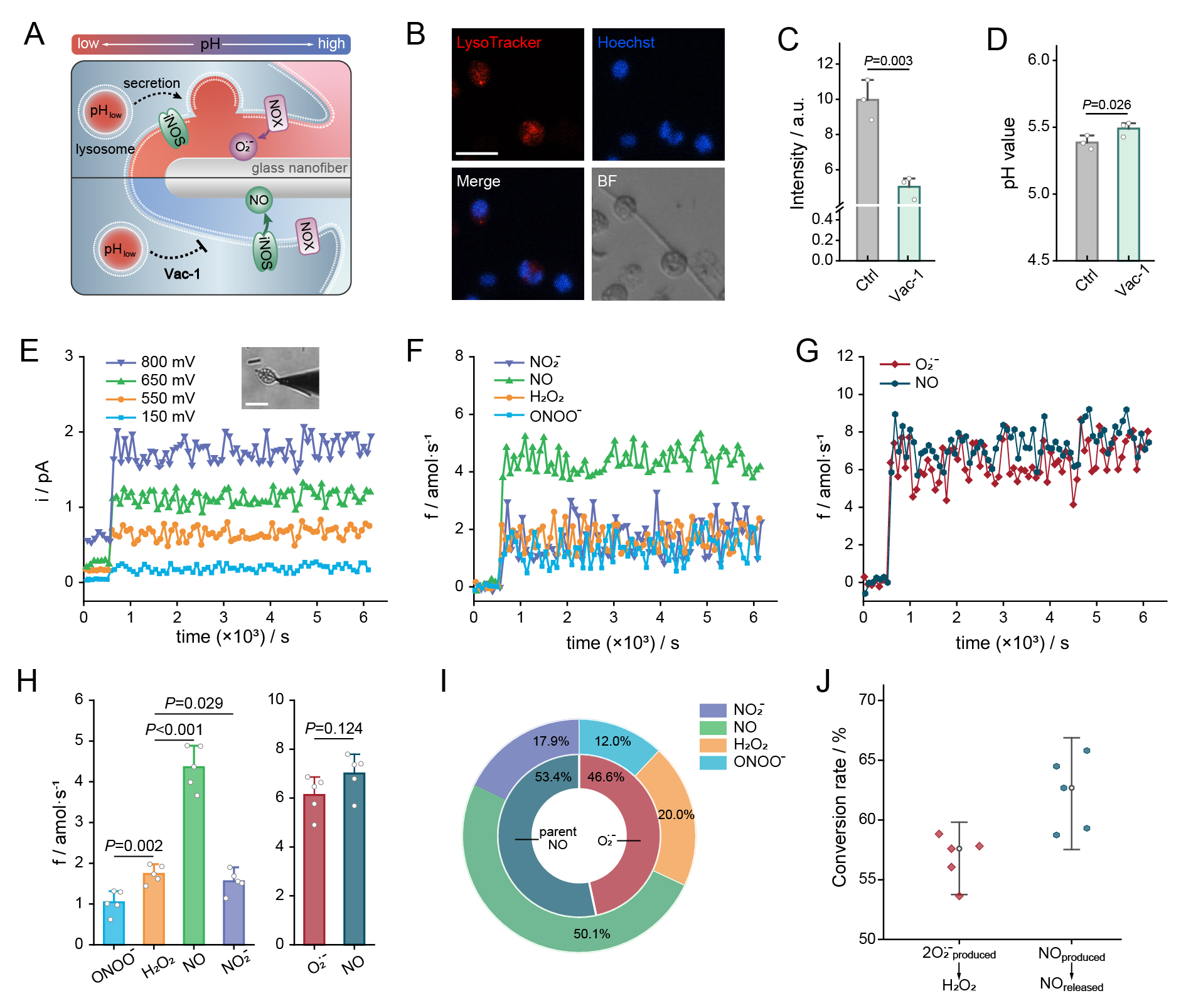
**

**Fig. S3. Intra-Lysosomal pH and ROS/RNS production of macrophage after 2 h Vacuolin-1 incubation after 10 h of frustrated phagocytosis. A)** Schematic representation of vacuolin-1 (Vac-1) alteration of the pH microenvironment of the phagocytic cup upon inhibiting lysosomal secretion. **B)** Morphology of macrophages stained with LysoTracker (red) and Hoechst dye (blue) and its bright-field image after 2 h incubation with 10 μM Vac-1. Scale bar, 20 μm. **C)** Statistical analysis (n = 90 cells from 3 independent samples) of the fluorescent intensity of stained LysoTracker with or without Vac-1 incubation (mean±SEM; one-way ANOVA). **D)** Intra-lysosomal pH value of macrophages with or without Vac-1 incubation (n= 3 tests, mean±SD.; one-way ANOVA). **E)** Time change of the chronoamperometric current measured at the end of each 20 s-staircase period at stepped potential values when the Pt NWS tip was near one cell/nanofiber junction point (inset photograph; scale bar, 20 μm). **F, G)** Corresponding time variations of production rates of four primary ROS/RNS **(F)** and their two parent precursors O_2_^•-^ and NO reconstructed fluxes **(G)**. **H)** Statistical analyses (n = 5 macrophages) of the production rates of the four primary ROS/RNS and their precursor (mean ± SEM; one-way ANOVA). **I)** Relative proportions of the four primary ROS/RNS and their two precursors O_2_^•-^ and NO as deduced from panel H. **J)** Conversion rates of H_2_O_2_ and released NO generated from their two parent precursors (O_2_^•-^_produced_ and NO_produced_) in light of the reaction stoichiometries illustrated in Fig. 2D.

**
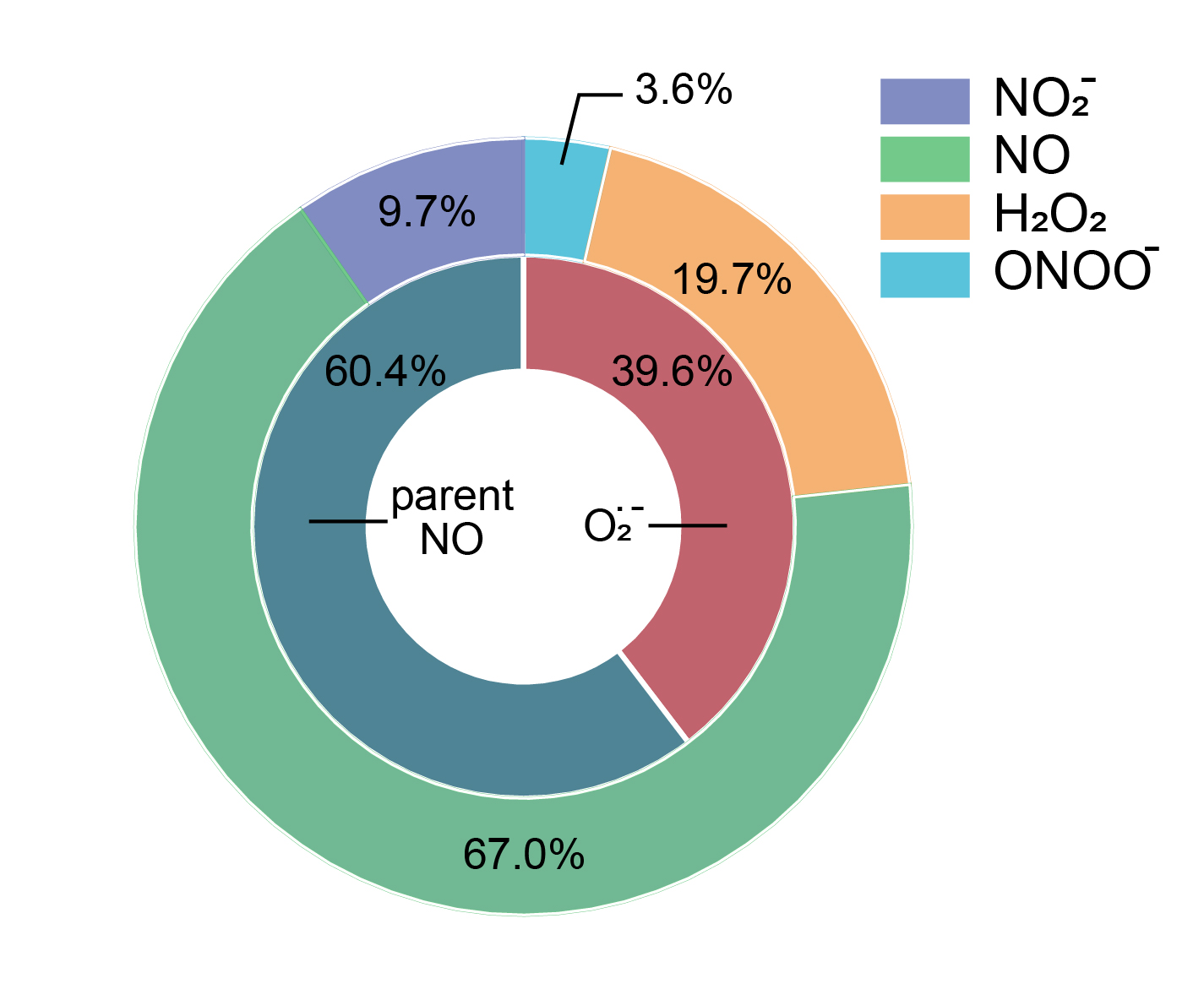
**

**Fig. S4. Proportion of ROS/RNS and their two parent precursors after 12 h of phagocytosis.** Relative proportions of the four primary ROS/RNS (ONOO^-^, H_2_O_2_, NO and NO_2_^-^) and of their two precursors (O_2_^•-^ and parent NO) following a 12-hour frustrated phagocytosis of glass nanofibers.


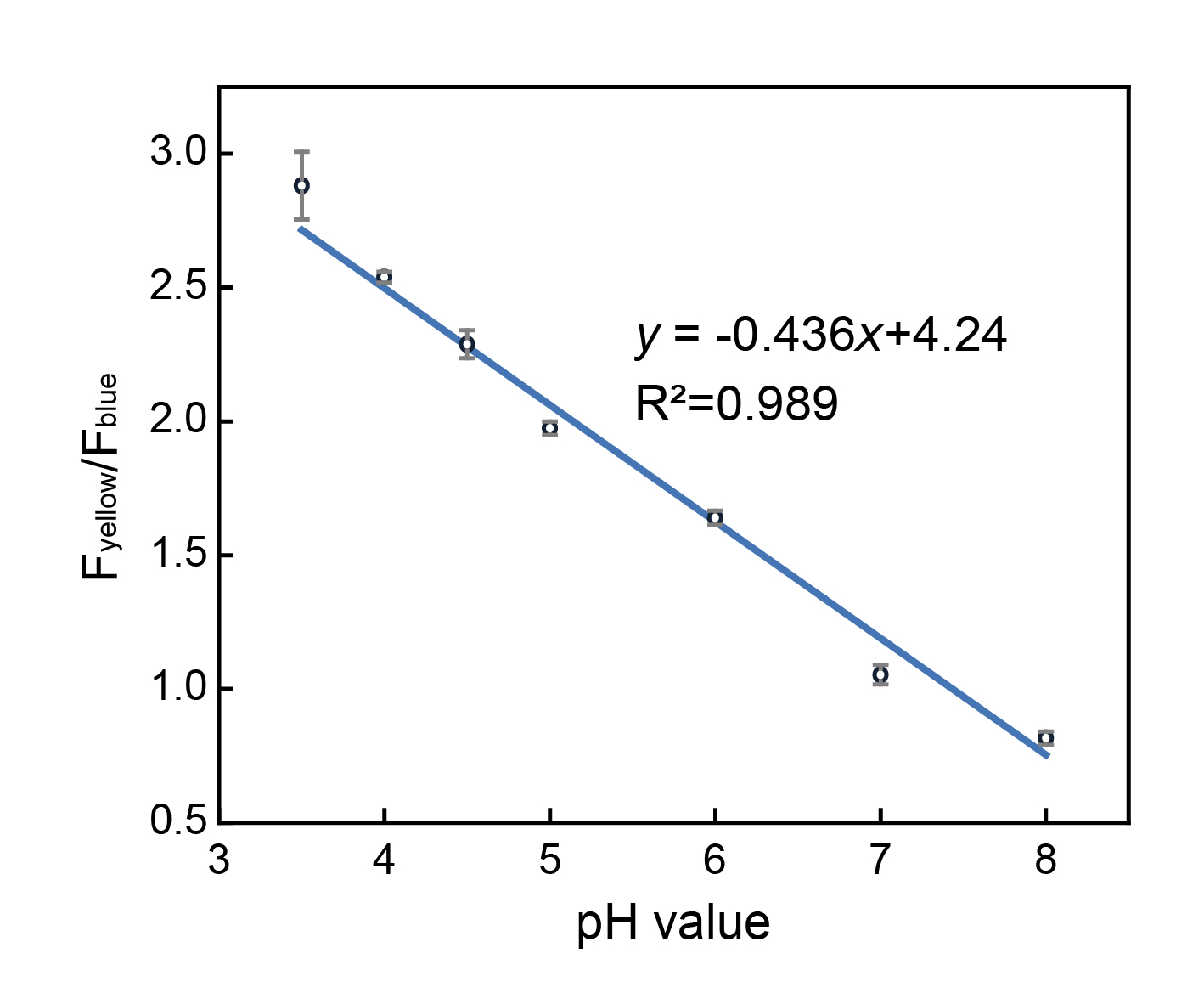


**Fig. S5. Standard calibration curve of the ratiometric dye LysoSensor Yellow/Blue DND-160.** See materials and methods section for experimental details.


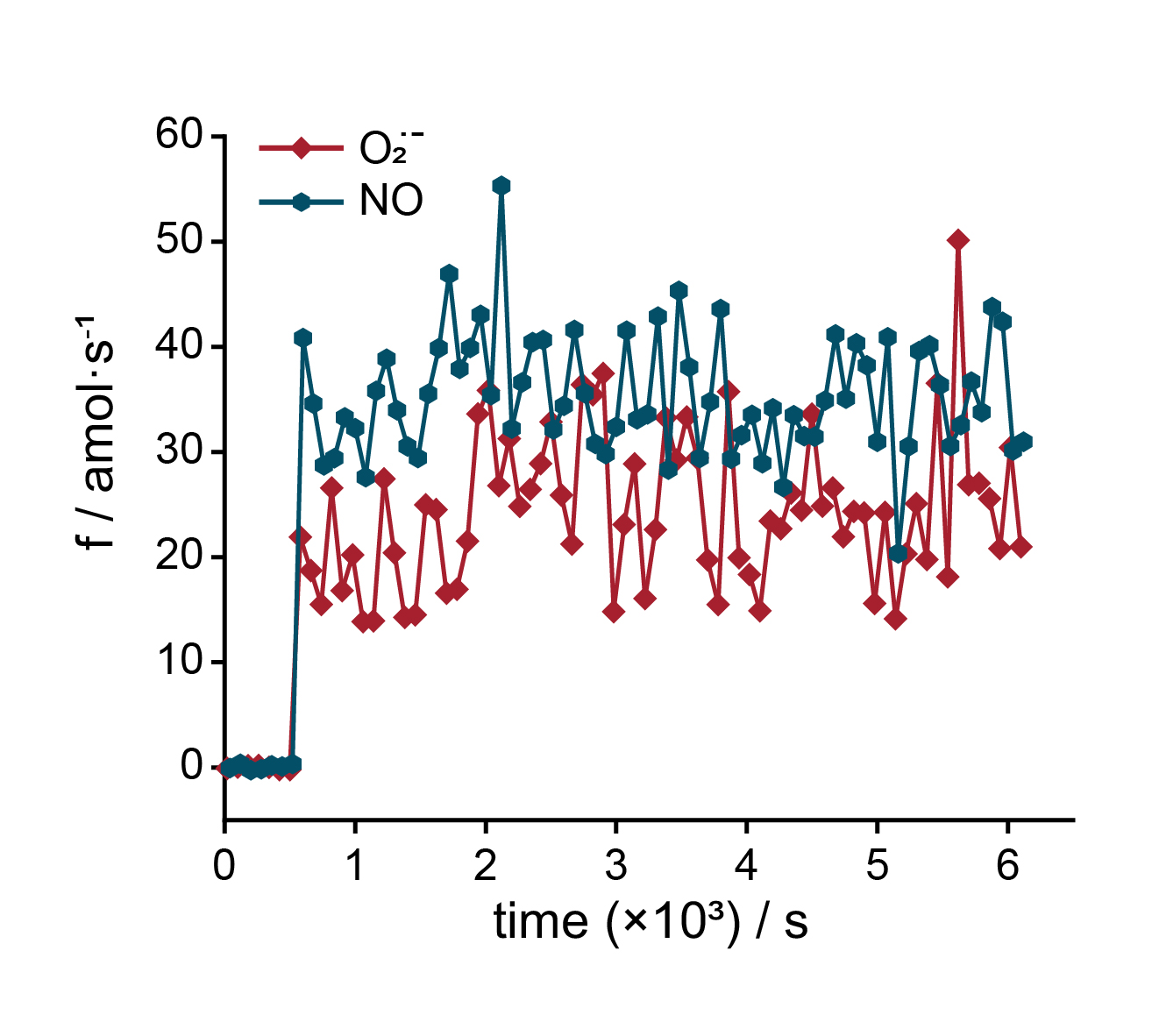


**Fig. S6.** Time variation of the production rates of the two precursors O_2_^•-^ and NO parents on the grounds of the four primary ROS/RNS amounts secreted at the phagocytic cup of macrophage following incubation with 100 μM EN6 for 2 h, after 10 h of frustrated phagocytosis.


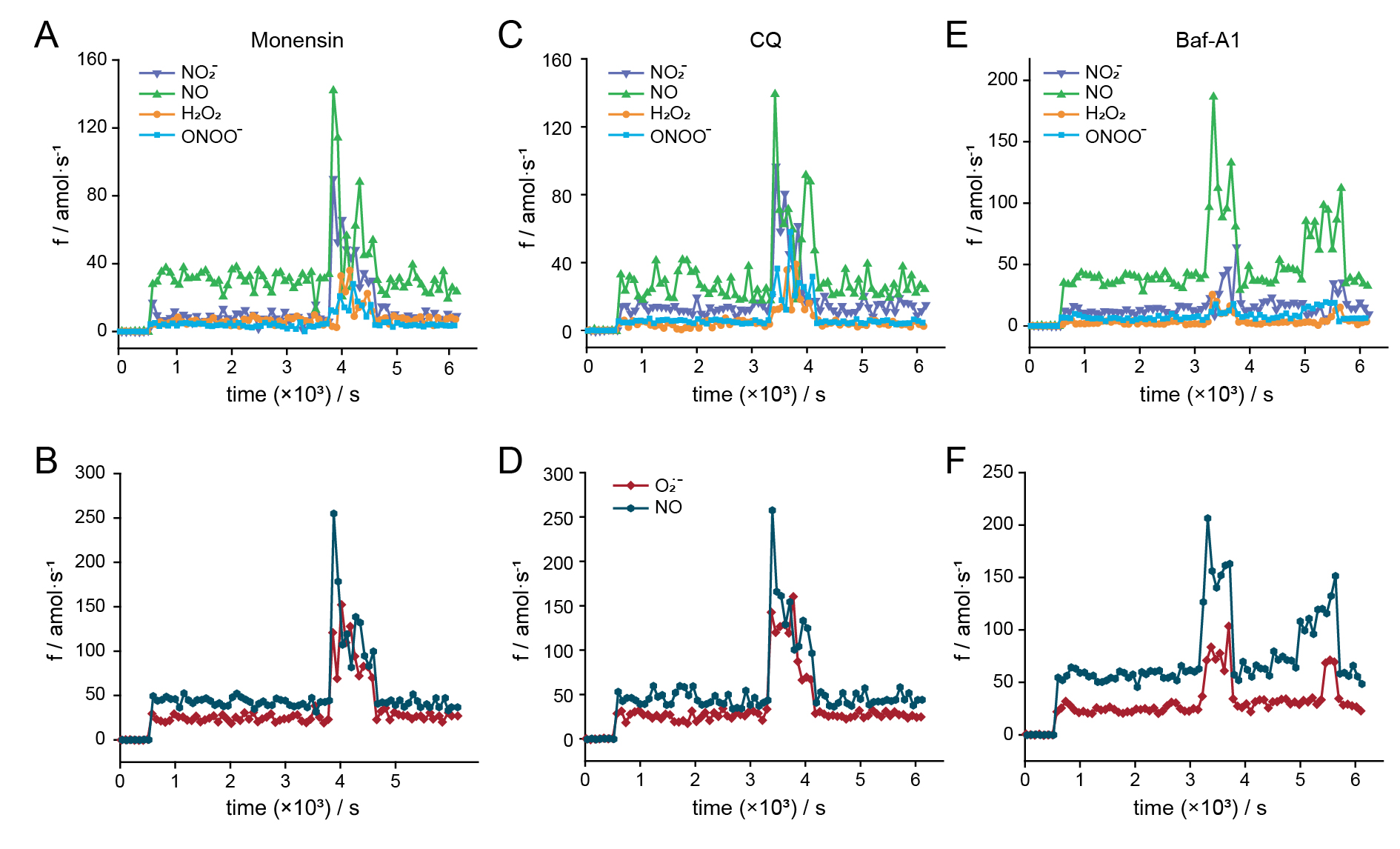


**Fig. S7. ROS/RNS production by macrophages after 2 h incubation with lysosomal alkalinizing regulators, after 10 h of frustrated phagocytosis. A, C, E)** Time changes of the production rates of ONOO, H_2_O_2_ NO and NO_2_^-^ at the phagocytic cup after incubation with 40 μM monensin (**A**), 20 nM CQ (**C**) and 100 nM Baf-A1 (**E**) for 2 h. **B, D, F)** Corresponding time variations of the reconstructed production rates of the two precursors O_2_^•-^ and NO according to **A, C, E** measurements at the phagocytic cup after incubation with monensin (**B**), CQ (**D**) and Baf-A1 (**F**).


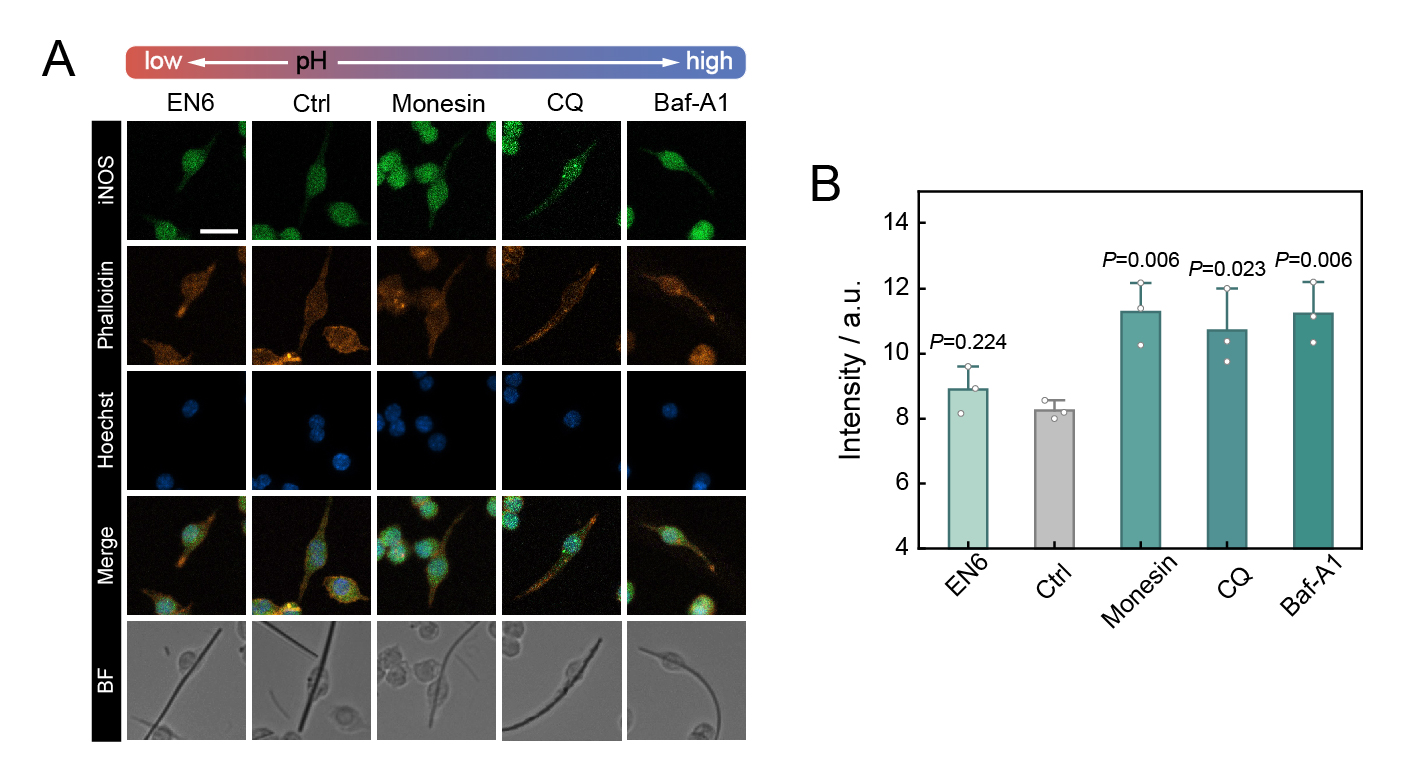


**Fig. S8. Expression of iNOS after 2 h incubation with or without lysosomal pH regulators, 12 h after beginning of frustrated phagocytosis.** **A)** Fluorescence images of macrophages stained for iNOS using its fluorescent-labelled monoclonal antibody (green), phalloidin (orange) and Hoechst (blue) after frustrated phagocytosis of glass nanofibers with or without lysosomal pH regulators incubation. **B)** Statistical analysis (n = 90 cells from 3 independent samples) of the fluorescent intensity of stained iNOS (mean±SEM; one-way ANOVA). Scale bar, 20 μm.

**
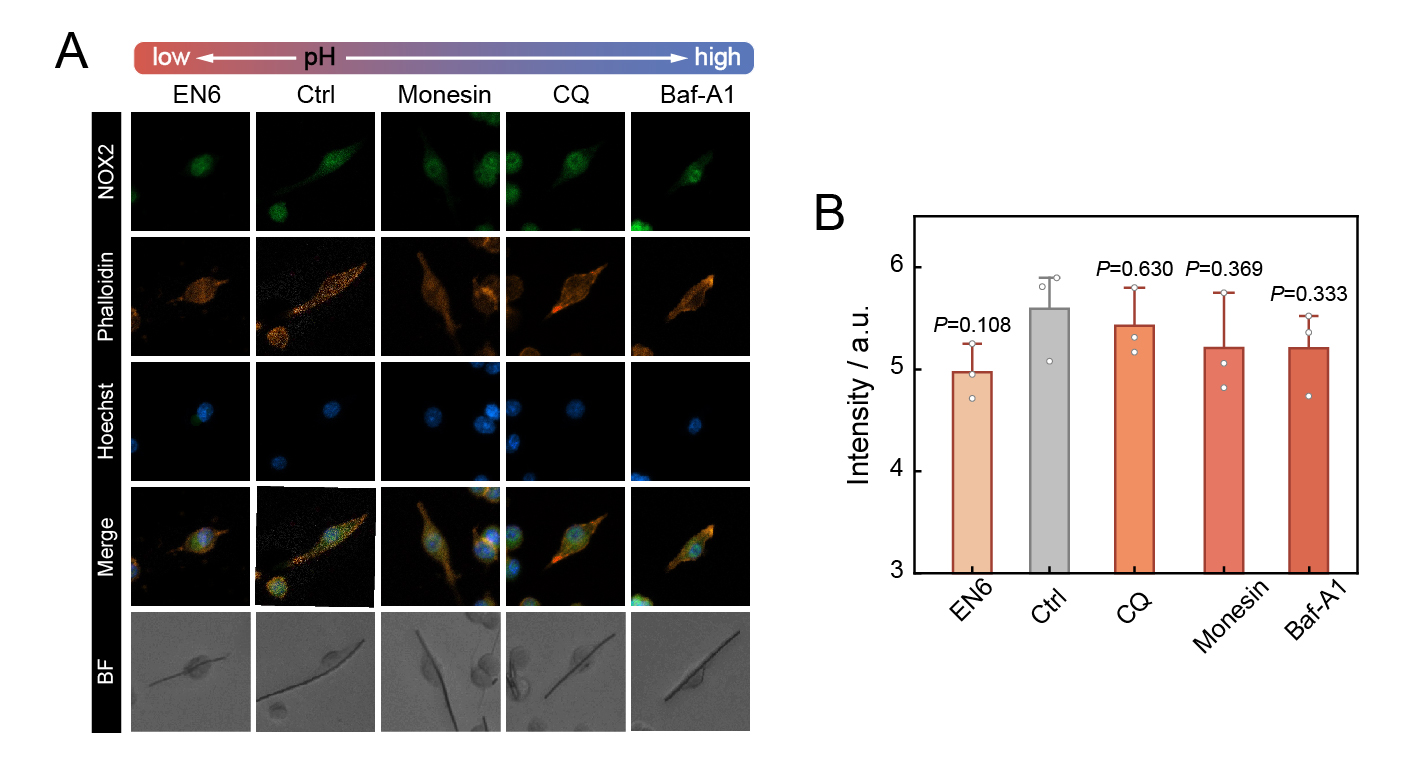
**

**Fig. S9. Expression of NOX2 after 2 h incubation with or without lysosomal pH regulators, 12 h after beginning of frustrated phagocytosis.** **A)** Fluorescence images of macrophages stained for NOX2 using its polyclonal antibody, FITC conjugated secondary antibody (green), phalloidin (orange) and Hoechst (blue) after frustrated phagocytosis of glass nanofibers with or without lysosomal pH regulators incubation. **B)** Statistical analysis (n = 90 cells from 3 independent samples) of the fluorescent intensity of stained NOX2 (mean±SEM; one-way ANOVA). Scale bar, 20 μm.


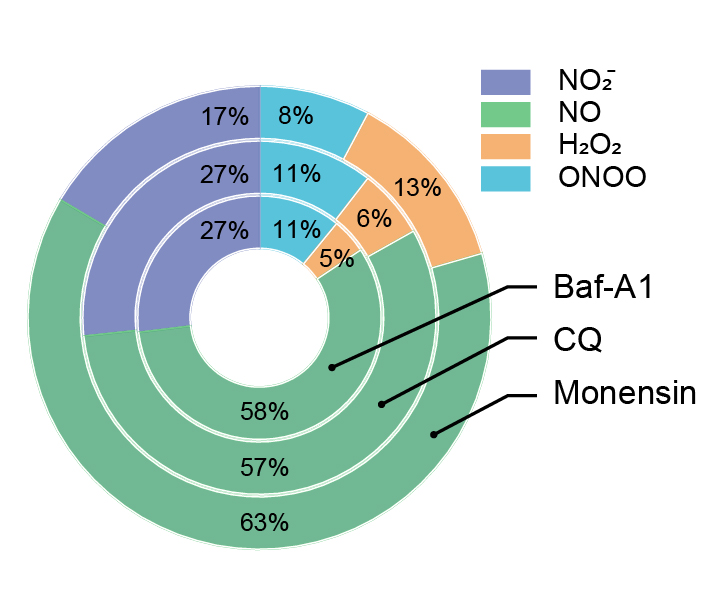


**Fig. S10. Relative proportions of the four primary ROS/RNS produced by macrophages after 2 h incubation with lysosomal alkalinizing regulators, after 10 h of frustrated phagocytosis.**

**SUPPLEMENTARY TABLES**

**Table S1.** Quantitative ROS/RNS production rates by macrophages at the phagocytic cup after frustrated phagocytosis of glass nanofibers within 12 hours with or without 2 h incubation of lysosomal pH regulators.

|  | **production rate / amol·s^-1^** | | | | | |
| --- | --- | --- | --- | --- | --- | --- |
|  | **ONOO^-^** | **H_2_O_2_** | **NO** | **NO_2_^-^** | **parent O_2_^•-^** | **parent NO** |
| **Ctrl** | 1.6±0.4 | 8.7±0.6 | 29.5±1.9 | 4.3±0.3 | 23.2±1.7 | 35.4±1.8 |
| **EN6** | 0.7±0.1 | 11.3±1.3 | 32.1±1.0 | 2.8±0.5 | 25.6±1.7 | 35.6±1.2 |
| **Monensin** | 3.8±0.2 | 6.2±0.4 | 30.6±1.1 | 8.0±0.7 | 24.1±1.2 | 42.4±1.0 |
| **CQ** | 5.1±0.4 | 3.1±0.7 | 27.3±3.0 | 12.9±1.0 | 24.5±1.5 | 45.3±3.0 |
| **Baf-A1** | 6.5±0.3 | 2.8±0.3 | 34.2±3.1 | 16.1±1.6 | 28.2±1.6 | 55.8±4.3 |
| **Vac-1** | 1.0±0.3 | 1.7±0.2 | 4.4±0.5 | 1.6±0.3 | 6.1±0.8 | 7.0±0.8 |

**Table S2.** Percentages of initially produced O_2_^•-^ and NO converted into H_2_O_2_ and released NO by macrophages after frustrated phagocytosis of glass nanofibers within 12 hours with or without 2 h incubation of lysosomal pH regulators.

|  | **conversion rate*^a^* / %** | |
| --- | --- | --- |
|  | **O_2_^•-^ _produced_→ H_2_O_2_** | **NO_produced_→ NO_released_** |
| **Ctrl** | 74.7±1.6 | 83.4±1.8 |
| **EN6** | 88.3±4.9 | 90.4±1.2 |
| **Monensin** | 51.4±2.0 | 72.1±1.5 |
| **CQ** | 25.2±4.5 | 60.2±2.8 |
| **Baf-A1** | 19.9±2.1 | 61.6±4.6 |
| **Vac-1** | 56.8±2.0 | 62.2±3.1 |

***^a^***Conversion rates represent the percentage of two precursors (O_2_^•-^ and parent NO) that ultimately yield H_2_O_2_ and NO in accordance with the conversion relationships of red (O_2_^•-^ _produced_→ H_2_O_2_) and blue (NO_produced_→ NO_released_) lines as illustrated in Fig. 2D.
